# Mating Increases CHST10 Activity in Rat Oviductal Mucosa to Induce the Synthesis of HNK-1 Glycoproteins: Possible Role in Sperm–Oviduct Interactions

**DOI:** 10.3390/ijms26073309

**Published:** 2025-04-02

**Authors:** Francisca Fábrega-Guerén, Juan C. Andrade, Marlene Zúñiga-Cóndor, Patricio Morales, Benito Gómez-Silva, Lidia M. Zúñiga

**Affiliations:** 1 Laboratorio de Bioquímica, Departamento Biomédico, Facultad de Ciencias de la Salud, Centre for Biotechnology and Bioengineering (CeBiB), Universidad de Antofagasta, Antofagasta 1271155, Chile; francisca.fabrega@uantof.cl (F.F.-G.); benito.gomez@uantof.cl (B.G.-S.); 2 Doctorado en Ciencias Aplicadas, Mención Sistemas Acuáticos, Ciencias del Mar y Recursos Biológicos, Universidad de Antofagasta, Antofagasta 1271155, Chile; 3 Laboratorio de Parasitología Molecular, Departamento de Tecnología Médica, Facultad de Ciencias de la Salud, Universidad de Antofagasta, Antofagasta 1271155, Chile; juan.andrade@uantof.cl; 4 Unidad de Citometría y Microscopía, Instituto Antofagasta, Universidad de Antofagasta, Antofagasta 1271155, Chile; marlene.zuniga@uantof.cl; 5 Laboratorio de Biología de la Reproducción, Departamento Biomédico, Facultad de Ciencias de la Salud, Universidad de Antofagasta, Antofagasta 1271155, Chile; patricio.morales@uantof.cl

**Keywords:** mating, oviduct, oviductal mucosa, CHST10, HNK-1 glycoproteins, sperm–oviduct interactions

## Abstract

Previously, we reported that mating induces an early transcriptional response in the oviductal mucosa of rats. The functional category ‘cell-to-cell signaling and interaction’ was overrepresented in this gene list. Therefore, in the present study, we describe the role of one of these genes, *carbohydrate sulfotransferase 10* (*Chst10*), in the oviductal mucosa. CHST10 participates in the synthesis of the carbohydrate moiety human natural killer-1 (HNK-1), which mediates cell-to-cell interactions. When using one-dimensional Western blot and sulfotransferase analyses, we found that mating increased the protein level and activity of CHST10 in the oviductal mucosa at 3 h after stimulation. A two-dimensional Western blot analysis and mass spectrometry were used to identify the novel HNK-1 glycoproteins aldehyde dehydrogenase 9 family, member A1 (ALDH9A1), fructose bisphosphate aldolase A (ALDOA), and four and a half LIM domains protein 1 (FHL1) in the oviductal mucosa, and we found that mating induces the synthesis of their acidic variants. Interestingly, in the utero-tubal junction (UTJ), acrosome-reacted sperm apparently were interacting with regions in which ALDH9A1 and HNK-1 signals overlap. Furthermore, vaginocervical stimulation applied to unmated rats increased the mRNA level of *Chst10* in the oviductal mucosa. In conclusion, mating increases the activity of CHST10 in the oviductal mucosa, which in turn induces the synthesis of acidic variants of ALDH9A1 and FHL1 via HNK-1 glycosylation. ALDH9A1, HNK-1-ALDH9A1, and/or other HNK-1 glycoproteins could participate in the negative selection of sperm in the UTJ, since we detected acrosome-reacted sperm apparently interacting with regions where these proteins are located. Finally, the sensorial component of mating could regulate early events (e.g., sperm transport and selection) occurring in the oviductal mucosa after mating.

## 1. Introduction

Mating induces physiological changes in the female reproductive tract, which regulate reproductive function. Sperm-independent components of mating, such as sensorial stimulation of the vaginocervical area and seminal plasma, modify the physiology of reproductive organs at various distances from the site of insemination. Recently, we reported that mating induces an early transcriptional response in the oviductal mucosa of rats [1], and we revealed that the signals that partially regulate this transcriptional response include tumor necrosis factor and retinoic acid. The cohort of mating-related genes was involved in cell-to-cell signaling and interactions; thus, in the present study, we describe the role of one of these genes, *carbohydrate sulfotransferase 10* (*Chst10*).

Glycoconjugates in the female reproductive tract are critical for controlling sperm maturation, sperm transport, and gamete interactions [2,3,4]. Thus, it is interesting that mating increases the transcriptional activity of *Chst10*, a gene encoding a carbohydrate sulfotransferase that participates in the synthesis of the human natural killer-1 (HNK-1) carbohydrate moiety. The structure of the HNK-1 moiety in glycoproteins and glycolipids is unique and consists of a sulfated trisaccharide with the sequence SO_4_-3GlcAβ1-3Galβ1-4GlcNAc–R [5]. The key enzymes involved in the biosynthesis of the HNK-1 moiety are β1,3-glucuronyltransferase [6,7], which transfers glucuronic acid (GlcA) in the β1→3 linkage to a terminal galactose of N-acetyl-lactosamine, and CHST10 sulfotransferase, which is responsible for coupling sulfate to the C-3 position of this GlcA residue [8,9]. The HNK-1 moiety mediates interactions among cells in the nervous system and immune system [10,11,12]; here, we report that it could mediate cell interactions in the reproductive system.

*Chst10* is upregulated in the oviductal mucosa of rats 3 h after mating [1]. This sulfotransferase is located in the Golgi apparatus, and in addition to its involvement in the synthesis of HNK-1 glycoproteins, it also participates in the generation of proteoglycans, specifically transferring sulfate to GlcA in chondroitin sulfate (CSA) [13,14,15]. Therefore, this sulfotransferase could modify the expression patterns of HNK-1 glycoproteins and/or proteoglycans, which must be exposed on the epithelial membrane and/or secreted into the oviductal lumen. Indeed, high Golgi apparatus activity and high sulfated glycoprotein secretion have been reported in response to mating in sheep and rabbits, respectively [16,17]. On the other hand, CHST10 is also involved in estrogen metabolism because it actively sulfates glucuronidated steroid hormones, preferring GlcA at the 3-hydroxyl group of a sterol ring over the 17-hydroxyl group [18]. CHST10 does not transfer sulfate to steroids lacking GlcA [18], which is a differential characteristic compared with that of cytosolic sulfotransferases (SULTs). In humans, three families of SULTs have been characterized: SULT1, SULT2, and SULT4; the SULT1E1 subfamily is specific for estrogens and sulfonate its 3-hydroxyl group [19]. CHST10 deficiency leads to high levels of estradiol (E_2_) in the serum, uterine hypertrophy, and infertility in mice [18]; therefore, this sulfotransferase has an important role in estrogen metabolism in vivo. In our rat model, mating changes not only the mechanism by which E_2_ accelerates oviductal egg transport but also the sensitivity to E_2_. Indeed, accelerating oviductal egg transport in mated rats requires a dose of E_2_ that is 10 times greater than that needed in unmated rats (10 µg/rat and 1 µg/rat, respectively) [20]. Therefore, exploring the specific processes regulated by CHST10 in oviductal physiology early after mating is imperative.

To achieve our objective, we measured the protein levels and activity of CHST10 in the oviductal mucosa, determined the localization of HNK-1 carbohydrate moieties in the oviduct, identified the HNK-1 glycoproteins present in the oviductal mucosa, and determined their localization. Finally, we identified the component of mating that regulates the expression of *Chst10* in the oviductal mucosa.

## 2. Results

### 2.1. Mating Increases CHST10 Protein Levels and Sulfotransferase Activity in the Oviductal Mucosa

**Figure 1:**
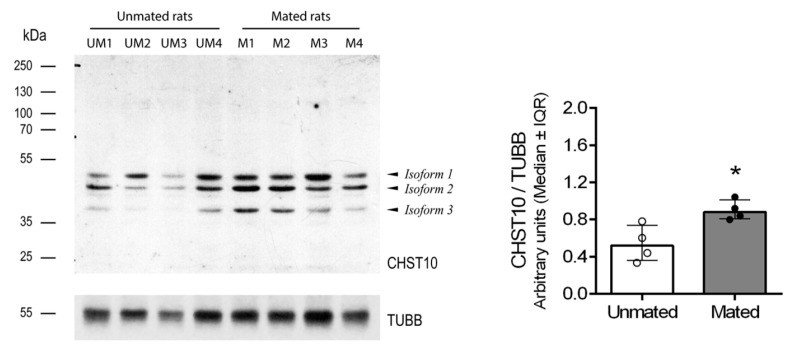
**Mating increases the carbohydrate sulfotransferase 10 (CHST10) protein level in the oviductal mucosa of rats.** Western blot of samples obtained from the oviductal mucosa of unmated (UM1-4) and mated (M1-4) rats. Biological replicates for each group, *n* = 4. The bar graph represents the intensities of the three CHST10 isoforms detected by the Western blot and normalized to that of tubulin β class I (TUBB). Interquartile range (IQR): Interquartile range. * *p* < 0.05, indicates significant differences.

**Figure 2:**
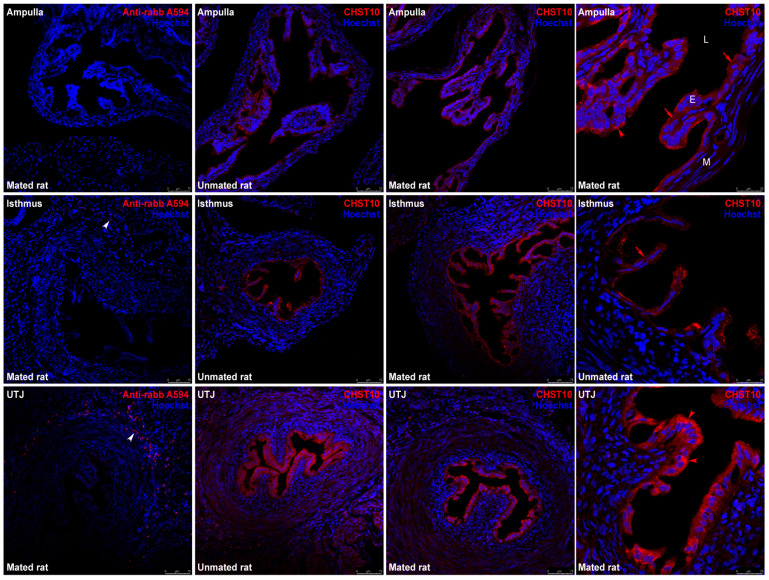
**Expression of CHST10 in the rat oviduct.** CHST10 was detected via immunofluorescence in oviductal sections obtained from unmated and mated rats. The panels show merged red and blue fluorescence. CHST10: red; and nuclear staining: blue. (**First row**) ampulla; (**second row**) isthmus; and (**third row**) utero-tubal junction (UTJ). The panels in the left column are the negative controls. White arrowheads: immune cells in negative controls; red arrowheads: CHST10 signal resembling the Golgi apparatus location; red arrows: CHST10 in the epithelium cytoplasm. L, Lumen; E, mucosa layer; M, muscular layer. Original magnification, 200× (scale bar, 75 μm). Only the right panels display the image at 630× magnification (scale bar, 25 μm). Images are representative of three independent experiments.

Mating increased the mRNA level of *Chst10* in the oviductal mucosa [1]; consequently, we measured CHST10 protein level by Western blotting to determine whether they affect oviductal physiology. As shown in Figure 1, we detected three bands in the oviductal mucosa from the unmated and mated rats, which corresponded to the CHST10 isoforms that have been proposed to be expressed in rats (see Discussion). We found that mating increased the protein levels of CHST10, considering the intensity of the three isoforms detected in the oviductal mucosa. Additionally, CHST10 was detected in the epithelium cytoplasm (Figure 2, fourth column, red arrows) of the ampulla, isthmus, and utero-tubal junction (UTJ); in some mucosal folds, the pattern of the detected signal resembled the location of the Golgi apparatus, especially in the UTJ (Figure 2, fourth column, red arrowheads). CHST10 was also expressed in the UTJ muscular layer (Figure 2).

**Figure 3:**
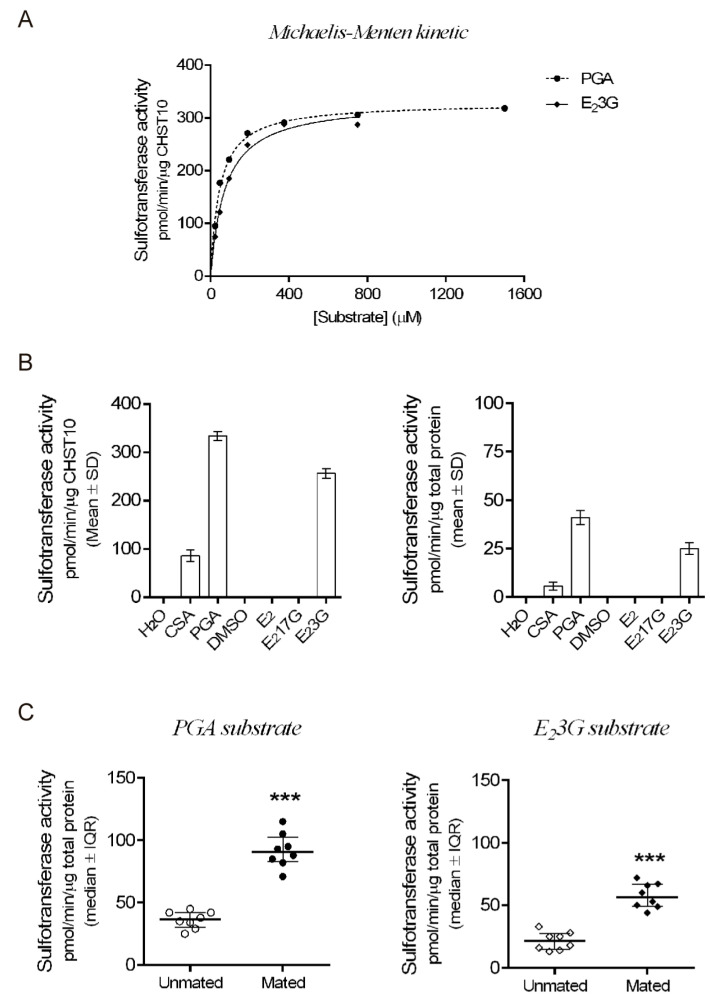
**Mating increases CHST10 activity in the oviductal mucosa of rats.** (**A**) Michaelis-Menten kinetics of human recombinant CHST10 to evaluate specific acceptor substrates. PGA: phenolphthalein glucuronic acid; E_2_3G: 17β-estradiol 3-[β-D-glucuronide]. Biological replicates for each group, *n* = 3. (**B**) Pattern of acceptor substrate preferences for CHST10 (**left**) and total protein from the oviductal mucosa of unmated rats (**right**). CSA: chondroitin sulfate A; E_2_: 17β-estradiol; E_2_17G: 17β-estradiol 17-[β-D-glucuronide]; DMSO: dimethyl sulfoxide. Biological replicates for each group, *n* = 3. (**C**) Plots of sulfotransferase activity measured in the oviductal mucosa of unmated and mated rats using PGA and E_2_3G as acceptor substrates. Biological replicates for each group, *n* = 8. *** *p* < 0.001, indicates significant differences.

Next, we measured the sulfotransferase activity in the oviductal mucosa. First, we determined the kinetic parameters of human recombinant CHST10 using two specific acceptor substrates (Figure 3A). CHST10 showed a preference for phenolphthalein glucuronic acid (PGA) [21], with K_m_ = 41.6 ± 2.4 μM and V_max_ = 328 ± 3.8 pmol/min/μg, compared with the K_m_ (73.9 ± 5.4 μM) and V_max_ (332 ± 7.1 pmol/min/μg) for 17β-Estradiol 3-[β-D-glucuronide](E_2_3G). Second, we compared the substrate preference patterns of CHST10 (Figure 3B, left) and the total proteins obtained from the cellular components of oviductal mucosa (Figure 3B, right). The substrate specificity patterns of CHST10 and the total proteins were similar, confirming that the activity measured in the oviductal mucosa corresponds to that of CHST10. Third, we measured sulfotransferase activity in oviductal mucosa extracts from unmated and mated rats using two specific acceptor substrates, PGA (Figure 3C, left) and E_2_3G (Figure 3C, right), and found that sulfotransferase activity in oviductal mucosa also increased because of mating; therefore, we further evaluated the expression of one of the CHST10-specific products in the oviduct, the HNK-1 carbohydrate moiety.

### 2.2. Mating Changes the Levels of HNK-1 Glycoproteins in the Oviductal Mucosa

**Figure 4:**
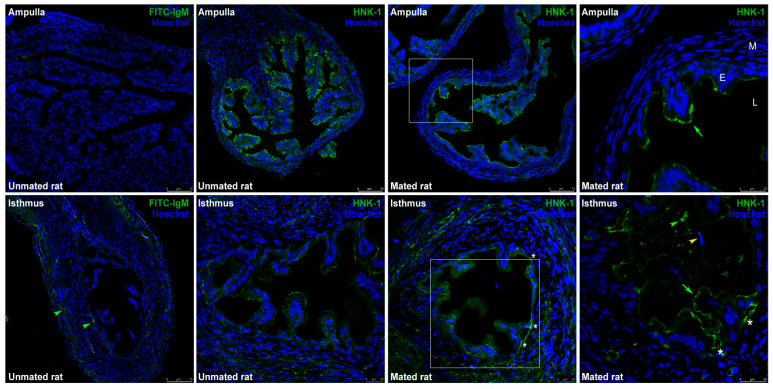
**Expression of human natural killer-1 (HNK-1) moieties in the rat oviduct.** Immunofluorescence images of the HNK-1 moiety in oviductal sections obtained from unmated and mated rats. The panels show merged green and blue fluorescence. HNK-1 moiety: green; nuclear staining: blue. (**First row**) ampulla; (**second row**) isthmus; original magnifications are 200× (scale bar, 75 μm) and 400× (scale bar, 25 μm). The square in the middle panels is the location of the magnified image displayed in the right panel, original magnification, 630× (scale bar, 25 μm). Green arrows: apical location of HNK-1 moieties in epithelial cells; green arrowhead: luminal location of HNK-1 moieties; yellow arrowhead: acrosome-intact sperm in the lumen; asterisks: HNK-1 moieties located in the trenches of the mucosal folds. L: lumen; E: mucosa layer; M: muscular layer.

CHST10 participates in the synthesis of the HNK-1 carbohydrate moiety, which is a glycolipid or glycoprotein prosthetic group. Immunofluorescence was used to detect the HNK-1 moiety in the oviduct, which is located on the luminal face of the epithelium. The signal in the ampulla was strong but weaker in the isthmus; thus, the isthmus images are shown at a high magnification (Figure 4). HNK-1 was also detected in the isthmus lumen (Figure 4, second row, fourth panel, green arrowhead); however, this signal was detected at a different Z-position than that in the image enclosed in the square in the second row, third panel. The signals detected in the muscular and serous layers of the isthmus were not specific since these signals were also detected in the isotype control (Figure 4, second row, first panel, green arrowheads).

**Figure 5:**
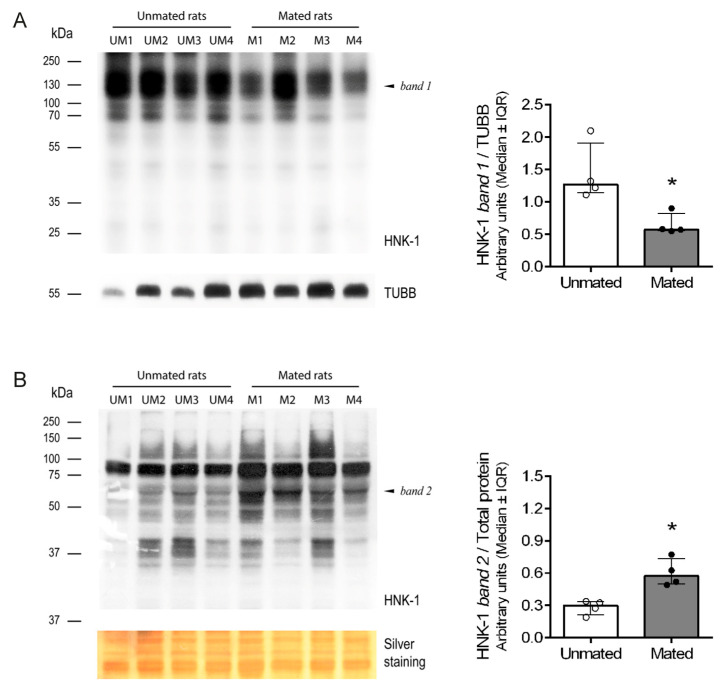
**Mating changes the levels of HNK-1 glycoproteins in the oviductal mucosa of rats.** (**A**) Western blotting via one-dimensional (1D) electrophoresis was used to evaluate HNK-1 glycoproteins in the *cellular pool* of proteins from the oviductal mucosa. Biological replicates for each group, *n* = 4. The bar graph presents the intensity of band 1 (130 kDa) normalized to that of TUBB. (**B**) Western blotting via 1D electrophoresis to evaluate HNK-1 glycoproteins in the *secreted pool* of proteins from the oviductal mucosa. Biological replicates for each group, *n* = 4. The bar graph shows the intensity of band 2 (55 kDa) normalized to that of the total proteins with molecular weights less than 37 kDa, as detected by silver staining of the nitrocellulose membrane. * *p* < 0.05, indicates significant differences.

Since mating increases the expression and activity of CHST10 in the oviductal mucosa, does mating also change the pattern of HNK-1 glycoprotein expression? Western blot analysis of the total proteins resolved by one-dimensional (1D) electrophoresis was conducted with samples obtained from the *cellular* and *secreted pools* of proteins from the oviductal mucosa. This approach revealed that mating decreased the intensity of the 130 kDa band in the *cellular pool* of glycoproteins (Figure 5A, left and right) while increasing the intensity of the 55 kDa band in the *secreted pool* of glycoproteins from the oviductal mucosa (Figure 5B, left and right).

**Figure 6:**
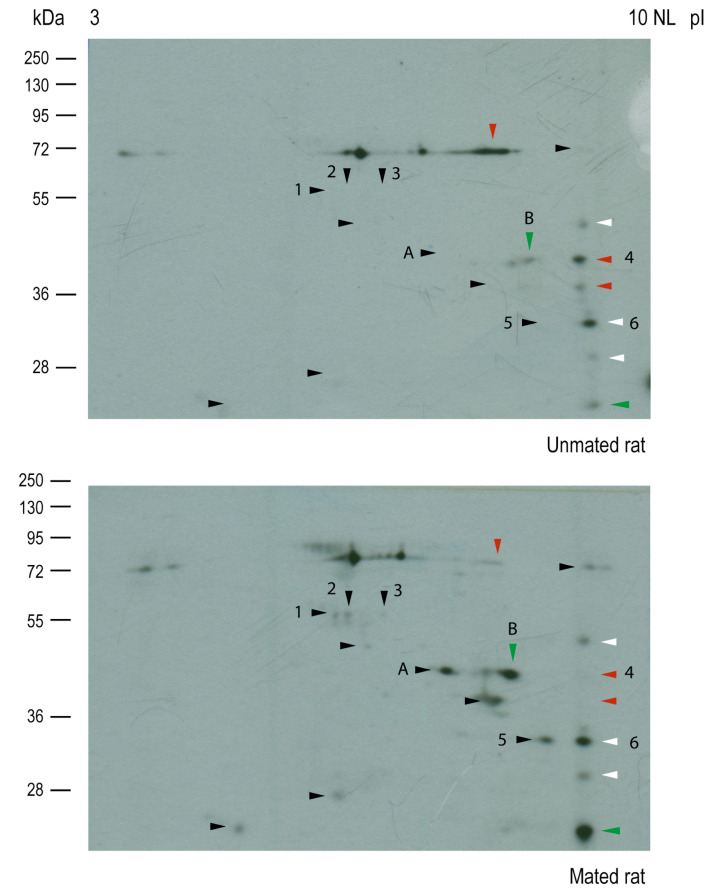
**Mating changes the levels of HNK-1 glycoproteins secreted from the oviductal mucosa of rats.** Western blotting via two-dimensional (2D) electrophoresis of samples from unmated (**upper**) and mated (**lower**) rats. Several HNK-1-positive spots were detected in both samples. Black arrowheads: mating-induced spots; green arrowheads: spots with increased intensities because of mating; red arrowheads: spots with decreased intensities because of mating; white arrowheads: spots with any change in intensity between samples.

Then, Western blot analysis of the samples resolved by two-dimensional (2D) electrophoresis was conducted to isolate and identify the HNK-1 glycoproteins (Figure 6); however, samples from the *cellular pool* were not well-resolved; then, we only focused on HNK-1 glycoproteins from the *secreted pool*. Our 2D analysis of HNK-1 glycoproteins revealed that mating changed the synthesis and/or secretion of a group of secreted HNK-1 glycoproteins in the oviductal mucosa. Our results indicated that mating induced the appearance of 10 spots (black arrowheads), increased the intensities of 2 spots (green arrowheads), and decreased the intensities of 3 spots (red arrowheads). Three spots with similar intensities were selected as references (white arrowheads) for sample comparison (Figure 6, upper and lower panels). Band 2 (55 kDa) previously detected in Figure 5B was resolved as spot 1, spot 2, and spot 3 and was detected only in mated rats (Figure 6, lower panel), supporting the results obtained after 1D electrophoresis.

**Figure 7:**
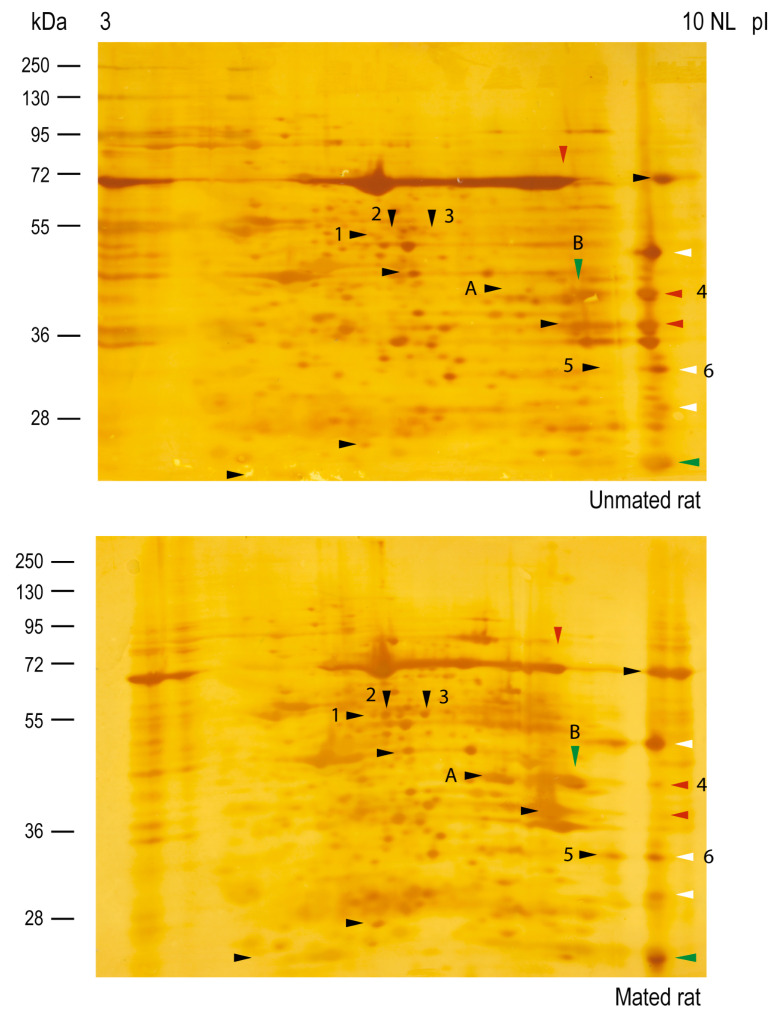
**Mating changes the protein *core* levels of HNK-1 glycoproteins secreted from the oviductal mucosa of rats.** Silver staining was performed on the membranes used for 2D Western blotting, as shown in Figure 6. Arrowheads: HNK-1 glycoproteins, colored arrowheads indicates changes for HNK-1 carbohydrate moiety previously described in Figure 6, note that, in almost all cases these changes corelate well with the protein *core* intensities detected by silver staining.

Silver staining of the nitrocellulose membranes used for the 2D Western blots revealed that the protein *cores* of spots 2, 3, 5, and A appeared only in samples from mated rats (Figure 7, lower panel), indicating that those proteins could be secreted and/or synthesized de novo. The intensities of the protein *cores* of spots 1 and 6 did not differ between samples, the intensity of the protein *core* of spot B increased in mated rats, and the intensity of the protein *core* of spot 4 decreased in mated rats. A new sample was subsequently analyzed via 2D electrophoresis and Coomassie blue staining, from which spots 2 and 3 were removed from the gel for mass spectrometry (MS) identification. We also identified spots 4, 5, and 6 since they were clearly resolved in the Coomassie blue-stained gel. The identified proteins are listed in Table 1. Spots 2 and 3 were identified as aldehyde dehydrogenase 9 family, member A1 (ALDH9A1), suggesting that they are isoelectric variants with different levels of HNK-1 glycosylation. Additionally, spots 5 and 6 were identified as four and a half LIM domains protein 1 (FHL1), whereas spot 4 was identified as fructose bisphosphate aldolase A (ALDOA).

### 2.3. Mating Induces the Secretion of ALDH9A1 and Increases the Secretion of ALDOA from the Epithelial Cells of Oviductal Mucosa

**Figure 8:**
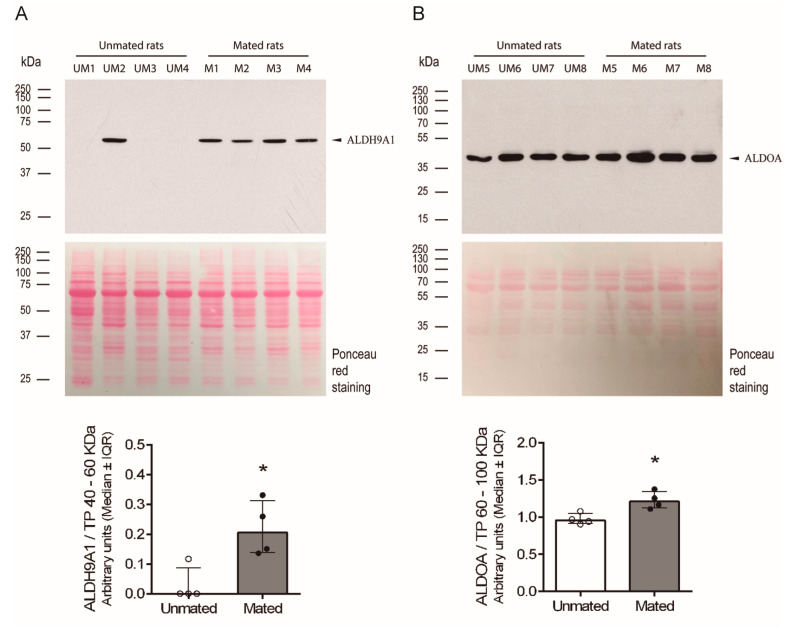
**Mating changes the levels of aldehyde dehydrogenase 9 family, member A1 (ALDH9A1) and fructose bisphosphate aldolase A (ALDOA) in the *secreted pool* of proteins from the oviductal mucosa of rats.** (**A**) Western blot analysis of ALDH9A1 expression in samples (40 µg each) obtained from the oviductal mucosa of unmated (UM1-4) and mated (M1-4) rats (upper panel). Biological replicates for each group, *n* = 4. The bar graph represents the intensity of the unique ALDH9A1 band (arrowhead) normalized to the total protein (TP) intensity between 40 and 60 kDa, as detected by Ponceau red staining of the nitrocellulose membrane (lower panel). (**B**) Western blot analysis of ALDOA expression in samples (10 µg each) obtained from the oviductal mucosa of unmated (UM5-8) and mated (M5-8) rats (upper panel). Biological replicates for each group, *n* = 4. The bar graph represents the intensity of the unique band (arrowhead) normalized to the TP intensity between 60 and 100 kDa, as detected by Ponceau red staining of the nitrocellulose membrane (lower panel). * *p* < 0.05, indicates significant differences.

After the proteins ALDH9A1 and ALDOA were identified, the above findings were validated by measuring the levels of these proteins in samples obtained from unmated and mated rats via 1D electrophoresis and Western blotting. Both proteins presented a unique band at the expected molecular mass, approximately 55 kDa and 40 kDa for ALDH9A1 and ALDOA, respectively (Figure 8A,B). We could not detect a specific band for the FHL1 protein. Since we do not know the identity of a secreted protein in these samples whose level was unchanged after mating, we applied Ponceau red stain to the total proteins on the Western blot membranes as a charge control to normalize the signal detected by the specific antibodies in the blot. The intensities of the protein bands between 40 and 60 kDa and between 60 and 100 kDa were used to normalize the ALDH9A1 and ALDOA signal intensities, since 40 and 10 μg of total protein per sample were used, respectively. Thus, mating induces the secretion of ALDH9A1 and increases the secretion of ALDOA from the epithelial cells of oviductal mucosa.

**Figure 9:**
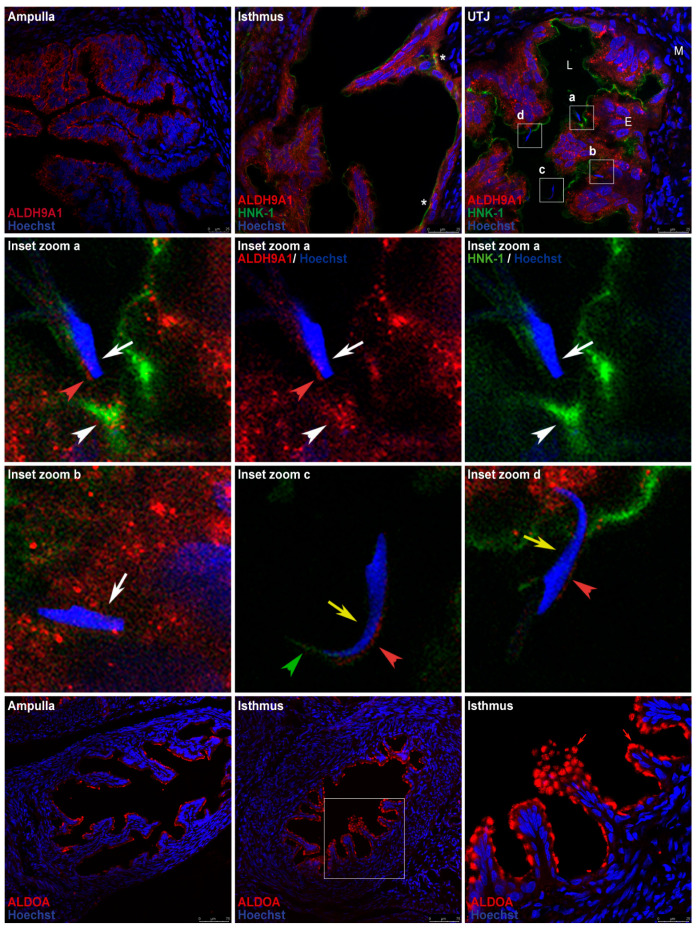
**Expression patterns of the HNK-1 glycoproteins ALDH9A1 and ALDOA in the oviductal mucosa of mated rats.** The HNK-1 moiety, ALDH9A1, and ALDOA were detected via immunofluorescence. In all panels, blue fluorescence corresponds to Hoechst nuclear staining. (**First row**) signals from ALDH9A1 (red) and HNK-1 (green). Asterisk: regional overlap of the HNK-1 and ALDH9A1 signals in the isthmus. UTJ: utero-tubal junction. Squares a, b, c, and d in the UTJ panel are the locations of the magnified images displayed in the second and third row. L, lumen; E, mucosa layer; M, muscular layer. (**Second and third rows**) Inset zoom images of sperm located in UTJ. White arrows: acrosome-reacted sperm (a and b); yellow arrows: acrosome-intact sperm (c and d); red arrowhead: weak signal of ALDH9A1; green arrowhead: weak signal of the HNK-1 moiety; white arrowheads: regional overlap of HNK-1 and ALDH9A1 signals. (**Fourth row**) ALDOA location (red). The square in the middle panel is the location of the magnified image displayed in the right panel. Red arrow: ALDOA localization resembling mucin vesicles; red arrowhead: apical location of ALDOA in epithelial cells. Original magnification, 200× (scale bar, 75 μm). The images in the panels in the right column and in the second row are shown at a magnification of 630× (bar, 25 μm). Images are representative of three independent experiments.

Immunofluorescence analysis of oviduct sections was used to localize ALDH9A1 and ALDOA in the oviductal mucosa. ALDH9A1 was detected on the luminal face of the epithelium in the ampulla and detected in the cytoplasm and cytoplasmic vesicles in the isthmus and UTJ. Double staining of ALDH9A1 (red signal) and the HNK-1 moiety (green signal) revealed that their signals were overlapping only in trenches created by the transverse mucosal folds in the isthmic region (Figure 9, first row, middle panel asterisk). Signal overlapping was also detected in the mucosa of the UTJ, closer to the membrane. We also found an acrosome-reacted sperm (Figure 9, second row, white arrow) that was apparently interacting with this signal-overlapping region in the mucosa (Figure 9, second row, white arrowhead), and another acrosome-reacted sperm (Figure 9, third row, left panel, white arrow) was found apparently crossing the oviductal mucosa. On the other hand, a free acrosome-intact sperm (Figure 9, third row, middle panel, yellow arrow) was detected in the lumen, and another acrosome-intact sperm (Figure 9, third row, right panel, yellow arrow) was apparently interacting with the oviductal mucosa, in a region in which the HNK-1 and ALDH9A1 signals did not overlap. Furthermore, we detected weak signals from ALDH9A1 in the acrosome’s principal segment domain of the acrosome-intact sperm (Figure 9, third row, middle and right panel, red arrowheads), and in the acrosome’s equatorial segment domain of the acrosome-reacted sperm (Figure 9, second row, left and middle panel, red arrowheads). Weak signals from HNK-1 moiety were only detected in the marginal segment domain of the acrosome in the free acrosome-intact sperm located in the oviductal lumen (Figure 9, third row, middle panel, green arrowhead). Finally, HNK-1 and ALDH9A1 weak signals were also detected in the middle piece of the sperm tail (Figure S1, green and red arrows, respectively). Additionally, tubulin β class I (TUBB), a marker of ciliated epithelia, was detected in the oviductal mucosa. Early after mating, ciliated epithelia are predominantly present in the ampulla, whereas they are scarce in the isthmus, mainly in the trenches created by the transverse mucosal folds, creating a striped pattern of ciliated cell clusters in the isthmus (Figure S2, right panel, green arrows).

Abundant ALDOA was detected in the luminal face of the epithelium in both the ampulla and the isthmus (Figure 9, fourth row, red arrowhead); in some mucosal folds, the pattern of the signal detected in the isthmus resembled mucin vesicles (Figure 9, fourth row, red arrow). Double staining of ALDOA and the HNK-1 moiety was not performed since the available ALDOA-specific antibody became inactivated during our experiments.

### 2.4. Vaginocervical Stimulation (VCS) Increases the Expression of Chst10 in the Oviductal Mucosa

**Figure 10:**
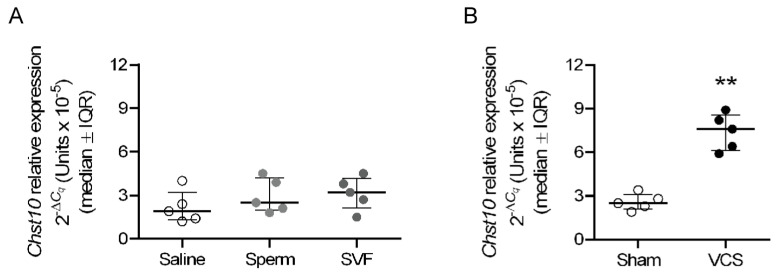
**Vaginocervical Stimulation (VCS) increases the level of *Chst10* in the oviductal mucosa of rats.** The graphs show the RT-qPCR results for *Chst10* normalized to the expression of reference genes. The scatter plots represent the normalized individual data points after 2^−ΔCq^ transformation for each individual sample. (**A**) Oviductal mucosa obtained 3 h after intrauterine injection of 0.1 mL of saline (0.9% *w*/*v* NaCl), a suspension of sperm in saline (10 million cells), or diluted seminal vesicle fluid (SVF, 1:1 dilution) into unmated rats. Biological replicates for each group, *n* = 5. (**B**) Oviductal mucosa obtained from unmated rats 3 h after VCS or sham stimulation. Biological replicates for each group, *n* = 5. ** *p* < 0.01, indicates significant differences.

To determine the mating-induced signal that regulates the expression of *Chst10* in oviductal mucosa, the immunological and sensorial components of mating were independently applied to unmated rats. In this study, 0.1 mL of saline, sperm suspension (10 million spermatozoa), or seminal vesicle fluid (SVF, diluted 1:1 in saline) was injected into the uterus to analyze the immune component of mating, and VCS or sham stimulation was performed to analyze the sensorial component of mating at 22:00 h of proestrus in unmated rats. Approximately 3 h after treatment (1:30 h), samples were obtained to determine the relative expression of *Chst10* via reverse transcription–quantitative polymerase chain reaction (RT-qPCR), with *Actin b* (*Actb*) and *glyceraldehyde-3-phosphate dehydrogenase* (*Gapdh*) used as reference genes. Finally, we found that only VCS increased the expression of *Chst10* in the oviductal mucosa of rats (Figure 10).

## 3. Discussion

Previously, we reported that mating induces an early transcriptional response in the oviductal mucosa of rats, indicating that the oviduct can organize a physiological response to sustain the events that occur in the oviduct early after mating. Here, we report one of those physiological responses.

Our results show that mating increases the activity of CHST10 and induces the synthesis of novel HNK-1 glycoproteins, which were identified as ALDH9A1, ALDOA, and FHL-1, in the oviductal mucosa. Three isoforms of CHST10 can be found in this tissue, with molecular masses ranging from 40 to 50 kDa. These bands correspond to CHST10 isoforms proposed to be expressed in rats (see the Rat Genome Databank: https://rgd.mcw.edu/rgdweb/report/gene/main.html?id=621216 (accessed on 6 January 2024)). Indeed, the sequence of the human antigen used to raise the antibody (Figure S3) has 81, 81, and 85% amino acid (aa) residue identity with CHST10 isoforms X1 (374 aas), X2 (356 aas), and X3 (288 aas), respectively (Figure S4A–C). The putative location of this sulfotransferase is the Golgi apparatus, and we detected signals suggesting this location, especially in the UTJ; however, we also detected CHST10 in the epithelium cytoplasm in all oviductal regions and in the muscular layer in the UTJ. Since estrogen sulfotransferases, such as SULT1E1, are located in the cytoplasm [19], we propose that cytosolic CHST10 can participate in estrogen metabolism in the oviductal mucosa. Indeed, in addition to its 3′-phosphoadenosine-5′-phosphosulfate (3′-PAPS) binding domain, CHST10 also contains a 5′-PAPS binding domain, which is characteristic of cytosolic sulfotransferases [19]. Moreover, we measured the specific sulfotransferase activity with glucuronidated estradiol in the oviductal mucosa. Therefore, we propose that in the oviductal mucosa, CHST10 participates not only in the synthesis of HNK-1 glycoproteins but also in estrogen metabolism. On the other hand, since we cannot discriminate which isoform exists in a particular subcellular location, we used the sum of the intensities of all the isoforms detected in each sample to compare CHST10 protein levels between unmated and mated rats. Thus, we found that mating increases the protein levels of CHST10 in the oviductal mucosa, which correlates with the measured increase in sulfotransferase activity when two specific acceptor substrates, PGA and E_2_3G, were used. Although we cannot test whether the sulfotransferase activity measured in the oviductal mucosa is exclusive to CHST10 due to a lack of specific inhibitors, we concluded that mating increases the activity of CHST10 in the oviductal mucosa, as supported by the following: (i) activity measurements with specific acceptor substrates (Figure 3C); (ii) a pattern of acceptor substrate preferences that is similar to that of human recombinant CHST10 (Figure 3B); and (iii) the endogenous production of its specific product, the HNK-1 carbohydrate moiety (Figure 4).

Compared with the isthmus and UTJ, the HNK-1 moiety is more abundant in the ampulla and is located predominantly on the luminal face of the oviductal epithelium. There was an unexpected diversity of HNK-1 glycoproteins in the oviductal mucosa, with clear differences in the molecular masses of the *cellular pool* (higher than 70 kDa) and the *secreted pool* (lower than 100 kDa) of glycoproteins (Figure 5). These results support the separation of glycoproteins into *cellular* and *secreted pools* by simple centrifugation after mechanical isolation of the oviductal mucosa and before lysis of its cellular components. However, in the *secreted pool* of glycoproteins, we cannot exclude the presence of some serum and/or cytosolic proteins released after the cells are damaged upon oviductal mucosal mechanical isolation. Our 2D analysis increased the power of HNK-1 glycoprotein detection. There were at least 15 HNK-1-positive spots with different intensities between the samples from unmated and mated rats, among which 5 were identified. Spots 2 and 3 were identified as ALDH9A1, as they have the same molecular mass but different isoelectric points. Although both are HNK-1 glycosylated proteins, spot 2 is more acidic than spot 3; therefore, spot 2 is the more densely glycosylated variant. Additionally, the protein *cores* of spots 2 and 3 were detected in samples from mated rats only (Figure 7, lower panel). Consequently, mating induces the de novo synthesis of HNK-1-ALDH9A1 isoelectric variants in the oviductal mucosa, and the presence of acidic variants could be strongly correlated with the increased activity of CHST10. We found similar results for spots 5 and 6, which were identified as FHL1. In this case, mating induces the de novo synthesis of the more acidic variant of FHL1 since spot 5 was detected only in samples from mated rats, whereas spot 6 was detected in both the mated and unmated samples. Therefore, mating induces the synthesis of acidic variants of specific HNK-1 glycoproteins in the oviductal mucosa. This conclusion could also be applied to ALDOA, although we only detected this protein in spot 4; however, we propose that spot A and spot B are acidic variants of ALDOA (Figure 6). We did not identify spot A or B because these spots were not well-resolved in the Coomassie blue-stained gels; that is, other spots were too close, as observed in the silver-stained membranes used for Western blotting (Figure 7). More importantly, contrary to our expectations, we cannot validate our initial interpretation of spot 4 from 2D analysis; that is, mating decreased its intensity (Figure 6 and Figure 7). Indeed, further Western blot analyses revealed that mating increased ALDOA levels (spot 4) in the *secreted pool* of glycoproteins from the oviductal mucosa (Figure 8B). This contradiction could be explained by the fact that spots A and B are acidic variants of ALDOA; indeed, spots A, B, and 4 have the same molecular mass, and spots A and B are located to the left of spot 4 (toward the acidic region). Furthermore, spot A was induced, and spot B was increased in mated samples (Figure 6 and Figure 7), which correlates well with the increased levels of ALDOA in the oviductal mucosa of mated rats. Overall, we conclude that mating increases the activity of CHST10 in the oviductal mucosa to induce the synthesis of acidic variants of ALDH9A1, FHL1, and ALDOA via HNK-1 glycosylation.

We propose that mating induces the de novo secretion of ALDH9A1 from the oviductal epithelium. Our results revealed that ALDH9A1 was not present in the oviductal mucosa-*secreted pool* of glycoproteins from unmated rats (except for one sample), but after mating, this glycoprotein was detected (Figure 8A). Similar results were detected by staining the 2D Western blot membrane with silver (Figure 7), since we did not detect an ALDH9A1 protein *core* (spots 2 and 3) in the unmated rat sample.

ALDH9A1 is located predominantly on the luminal face of the epithelium in the ampulla, but it is also found in the cytoplasm and cytoplasmic vesicles in the isthmus and UTJ. In the isthmus, double staining of ALDH9A1 (red signal) and the HNK-1 moiety (green signal) revealed that their signals were overlapping, specifically in the trenches created by the transverse mucosal folds (Figure 9, first row, middle panel asterisk), which could be related to the ciliated cell clusters also found in these trenches, creating a striped pattern of ciliated cell clusters in the isthmus (Figure S2, green arrows), as has also been observed in mice [22]. This striped pattern is like the pattern of sperm distribution in the mice isthmus, as shown previously [23,24,25]. Considering that ordinary confocal fluorescence microscopy cannot reliably detect signal colocalization because of signal diffraction effects and resolution limit, the merged yellow signal detected in our samples can be preliminarily proposed as evidence for the presence of HNK-1-ALDH9A1 glycoprotein, which has previously been identified by Western blot and MS in oviductal mucosa samples (Figure 6 and Table 1). We may conclude that the presence of HNK-1-ALDH9A1 glycoprotein at the signal-overlapping regions cannot be confirmed or excluded. Therefore, in these signal-overlapping regions, we can detect ALDH9A1, HNK-1-ALDH9A1, and/or other HNK-1 glycoproteins. On the other hand, the observed HNK-1 and ALDH9A1 signal overlapping was also detected at the UTJ (Figure 9, first row, right panel, square a), but in this case, it appeared closer to the membrane of the epithelium (Figure 9, second row, white arrowhead), which apparently was interacting with an acrosome-reacted sperm (Figure 9, second row, white arrow). We hypothesize that signal-overlapping regions in the UTJ could be involved in the negative selection of sperm since sperm could be induced to undergo acrosome reaction (AR) in those regions. In support of this hypothesis, we highlight the detection of an acrosome-intact sperm apparently interacting with the mucosa in a region where the HNK-1 and ALDH9A1 signals did not overlap (Figure 9, third row, right panel, yellow arrowhead).

The ALDH9 family encodes enzymes involved in the metabolism of 4-aminobutyraldehyde (ABAL) and amino aldehydes derived from polyamines and choline [26]. ALDH9A1, which catalyzes the irreversible NAD(P)-dependent oxidation of aliphatic or aromatic aldehydes, was first purified from the liver and was characterized as an ABAL dehydrogenase that forms γ-aminobutyric acid (GABA) [27], a well-known neurotransmitter; however, GABA is also an inducer of the AR in the sperm of many species in vitro [28,29,30,31]. Interestingly, the abundance of GABA in the rat oviduct is 2.5 times greater than that in the whole brain [32]. GABA is synthesized and secreted by the oviductal mucosa [33], and its concentration is greater at diestrus [32]. Here, we report that ALDH9A1 is highly expressed in the oviductal mucosa (Figure 9), which correlates well with GABA abundance; however, its physiological role in the oviduct in vivo remains to be determined. Recently, ALDH9A1 was shown to exhibit wide substrate specificity but has a clear preference for γ-trimethylaminobutyraldehyde (K_m_ = 6 ± 1 μM) [34]; other novel identified substrates include 4-guanidinobutyraldehyde (GBAL) and 3-aminopropanal (APAL), with K_m_ values of 21 ± 2 μM and 56 ± 7 μM, respectively [34]. APAL is produced during spermine metabolism by the sequential actions of spermine oxidase and polyamine oxidase [35]. Interestingly, spermine is a ubiquitous polyamine present at millimolar concentrations in the seminal plasma of many species, including humans, rats, and rams [36]. Spermine is rapidly incorporated into sperm during ejaculation and temporarily inhibits premature capacitation and the spontaneous acrosome reaction (sAR); indeed, spermine has been proposed as the major decapacitation factor in seminal fluid [37,38,39]. Therefore, considering that ALDH9A1 participates in spermine metabolism and GABA production, and that we detected an acrosome-reacted sperm apparently interacting with a region where ALDH9A1 and the HNK-1 moiety are located, we propose that ALDH9A1 in close contact with sperm could adjust the content of spermine in the sperm during their journey to the site of fertilization. Therefore, if sperm with a low content of spermine arrives at the UTJ and contacts ALDH9A1 regions, the spermine content will surpass the critical limit, and the sperm will be induced to undergo sAR or will be capacitated and undergo AR because of the GABA present in the oviductal mucosa.

On the other hand, in free sperm located in the oviductal lumen, we detected weak signals from ALDH9A1 and HNK-1 in the principal and marginal segment domains of the acrosome, respectively (Figure 9, third row, middle panel, red and green arrowheads). Both signals clearly delimited those regions and did not overlap. This finding is very interesting since molecules exposed on the rostral surface of the sperm head interact with glycoproteins from the oviductal epithelium [40], suggesting that the HNK-1 moiety on the sperm surface could mediate interactions with HNK-1 receptors on the surface of epithelial cells or in the extracellular matrix (ECM). The proposed receptors for the HNK-1 carbohydrate moiety are interleukin-6 [41], L and P-selectin [42], tenascin C (also called cytotactin) [43], laminin [44], and brevican [45], some of which have been reported to be expressed in the oviduct [46,47]. Interestingly, it has been reported that adam6-deficient mouse sperm cannot ascend into the oviduct because they cannot bind temporarily to ECM components such as fibronectin, laminin, and tenascin [48], which constitute an ascent route for the transition of sperm from the uterus to the oviduct, and here, we report free sperm arriving at the UTJ containing the ligand for these ECM glycoproteins. This is the first report showing the presence of the HNK-1 moiety on the marginal segment domain of the acrosome (Figure 9, third row, middle panel, green arrowhead) and middle piece of the tail (Figure S1A) in vivo, and to elucidate its sperm function, we must work with ejaculated sperm since we did not detect the HNK-1 moiety in sperm obtained from the cauda of the rat epididymis; therefore, we propose that the HNK-1 glycoprotein detected on the sperm surface could be provided by the seminal or the uterine fluid. This is also the first report showing the presence of ALDH9A1 on the sperm surface and/or in the sperm acrosome in vivo (Figure 9, second and third rows, red arrowheads); therefore, to elucidate its sperm function, first, we must elucidate whether this protein is synthesized by the sperm since the expression of other ALDHs has been reported [49].

Finally, sensorial stimulation of the vaginocervical area induces physiological processes in females necessary for reproductive success, such as ovulation in rabbits [50] and pseudopregnancy in spontaneous ovulators such as rats [51]. We showed that VCS increases the mRNA level of *Chst10* in the oviductal mucosa of rats. Our data suggest that, in the oviductal mucosa, an increase in *Chst10* transcript is correlated with increased CHST10 protein level and activity; therefore, we propose that all events described in this paper can be regulated by the sensorial component of mating. It is conceivable that signals regulating sperm-oviduct interactions must arrive at the oviduct earlier than sperm. Thus, the sensorial stimulation associated with mating possibly arrives at the oviduct via the neuronal network that connects the vagina to the oviduct [52], since sperm arrives in the oviduct 15 min after mating [53]. Therefore, our findings merit further research since they indicate that the sensorial component of mating can regulate events occurring in the oviductal mucosa early after mating.

## 4. Materials and Methods

### 4.1. Animals

We used 102 Sprague-Dawley rats, 73 females (240–280 g) and 29 males (400–600 g), in this study. The animals were kept under controlled environmental conditions (temperature of 21–24 °C and lights on between 7:00 and 21:00 h) and were provided rodent chow and water *ad libitum*. Under these controlled conditions, our animals ovulated at approximately 6:30 h of estrus. The estrus cycle was evaluated by performing daily vaginal smears, and all females were used after exhibiting two consecutive 4-day cycles.

#### 4.1.1. Mating and Sample Collection

Females in proestrus were isolated (unmated rats) or caged with fertile males at 22:00 h of proestrus (mated rats). Thirty minutes after being caged with the males, the presence of spermatozoa in a vaginal smear or a vaginal plug was verified. The unmated and mated rats were subsequently sacrificed at 1:30 h of estrus for oviduct excision and dissection. The oviductal mucosa was obtained under a stereomicroscope using curved microsurgical scissors and fine forceps. Briefly, the mesothelial membrane of the oviducts was removed in saline (0.9% NaCl) to compensate for oviductal folding. Then, the defolded oviducts were dried, placed on a new embryological culture dish with 0.4 mL of saline containing the antiprotease cocktail cOmplete (RocheMannheim, Germany, cat# 04693132001) at 4 °C, and cut into four pieces. Afterward, each piece was held with fine forceps on one side, and with another set of forceps, the piece was carefully extruded to obtain the mucosa. The mucosa pieces were collected with a micropipette and added to a 0.6 mL conical tube. After centrifugation at 3000× *g* for 10 min, the supernatant was collected in a new conical tube. The pellet contained both epithelial and stromal cells, whereas the supernatant contained proteins that had been secreted into the oviductal lumen, which we call the *secreted pool*. However, in this *secreted pool* of proteins, we cannot exclude the presence of serum and/or cytosolic proteins that are released when the cells are damaged due to mechanical isolation of the oviductal mucosa.

#### 4.1.2. Sperm and Seminal Vesicle Fluid (SVF) Collection

Male rats were sedated and sacrificed by decapitation at 21:30 h of female proestrus to obtain their epididymal spermatozoa and SVF. Each cauda epididymis was dissected in saline at 37 °C; then, several regions were cut open in 0.5 mL of saline overlaid with mineral oil at 37 °C to allow the sperm to diffuse into the medium [54]. The concentration of sperm in the suspension was determined using a Neubauer Chamber (Cambridge Instruments, London, UK). Concomitant with the previous procedure, we used a 1 mL syringe (BD Plastipak, Curitiba, Brazil, cat# 990214) with a 21G × 1 ½″ gauge (BD Plastipak, cat# 300340) to obtain the fluid from both seminal vesicles by inserting the gauge into the lumen of each seminal vesicle and aspirating the fluid. We obtained approximately 0.5 mL of fluid, which was diluted 1:1 with saline at 37 °C.

#### 4.1.3. Intrauterine Injection of Sperm or SVF

To identify the mating component that regulates *Chst10* expression in the oviductal mucosa, female rats were randomly distributed into 3 groups. Each group received one of the following via intrauterine injection at 22:00 h of proestrus: 0.1 mL of saline (*n* = 5), 0.1 mL of sperm suspension containing 10 million sperm (*n* = 5) in saline, or 0.1 mL of diluted SVF (*n* = 5). Surgical intervention was conducted as follows. First, the animals were anesthetized with 75 mg/kg ketamine + 5 mg/kg xylazine via intraperitoneal injection. The uteri were exposed by creating flank incisions, and 0.1 mL of saline, sperm suspension, or SVF was injected into the upper third of each uterine horn using a 1 mL syringe. Then, the uteri were returned to the peritoneal cavity, and the muscles and skin were sutured. The procedure lasted 15 min. Finally, the oviductal mucosa was obtained at 1:30 h of estrus and stored in TRIzol reagent (Ambion, Carlsbad, CA, USA, cat# 15596-018) at −80 °C.

#### 4.1.4. Vaginocervical Stimulation

To identify the mating component that regulates *Chst10* expression in the oviductal mucosa, female rats were randomly distributed into 2 groups. In the first group, females in proestrus were mechanically stimulated at the vaginocervical area at 22:00 h (*n* = 5). Mechanical stimulation was applied by introducing 1 cm of a glass vibrating rod into the vagina twice for 10 s each time with an interval of 5 s. The rats in the second group (*n* = 5) were manipulated following the same procedure as that used for the rats in the stimulation group but did not receive mechanical stimulation (sham control, *n* = 5). The procedure lasted 10 min. Finally, the oviductal mucosa was obtained at 1:30 h of estrus and stored in TRIzol reagent at −80 °C.

### 4.2. Sulfotransferase Activity

To measure sulfotransferase activity, we used both unmated and mated female rats. Sulfotransferase activity was measured using a Universal Sulfotransferase Activity Kit (R&D Systems, Minneapolis, MS, USA cat# EA003), and PAPS (R&D Systems, cat# ES019) was used as the donor substrate. PGA (Sigma-Aldrich, Saint Louis, MO, USA cat# P0501), E_2_3G (Cayman Chemical, Ann Arbor, MI, USA, cat# 16155), and CSA (Sigma-Aldrich, cat# C9819) were used as positive acceptor substrates, and 17β-estradiol 17-[β-D-glucuronide] (E_2_17G; Cayman Chemical, cat# 16156) and E_2_ (Cayman Chemical, cat# 10006315) were used as negative acceptor substrates. We also evaluated the substrate specificity of human recombinant CHST10 (R&D Systems, cat# 6140-ST) and compared the activity of this enzyme to the sulfotransferase activity detected in oviductal mucosa protein extracts.

The oviductal mucosa protein extract was obtained as follows. First, 150 µL of phosphatase 3 buffer (provided in the kit) was added to the oviductal mucosal epithelial and stromal cell pellet. Then, the sample was sonicated at power 20 for 15 s twice at 4 °C; afterward, the sample was centrifuged at 8000× *g* for 10 min at 4 °C, and the supernatant was stored at −20 °C for further analysis. This assay uses coupling phosphatase 3 (CP3) to remove inorganic phosphate (Pi) from the leaving PAP nucleotide, and the released Pi is detected by malachite green phosphate detection reagents. The amount of Pi released by CP3 is proportional to the amount of PAP generated during the sulfotransferase reaction; therefore, the rate of Pi production reflects the kinetics of the sulfotransferase reaction (R&D Systems, cat# EA003). A standard curve of Pi was generated following the manufacturer’s instructions, and the stability of PAPS to CP3 was evaluated in each experimental procedure. Sulfotransferase activity was evaluated by measuring the absorbance at 620 nm using an Infinite^®^ F50 microplate reader (TECAN Austria GmbH, Grödig, Austria) after 20 min of incubation at 37 °C. The total protein concentration in the oviductal mucosa protein extracts was quantified using the Bradford method (Bio-Rad, Hercules, CA, USA, cat# 500-0006) with bovine serum albumin (BSA; EMD Millipore, Billerica, MA, USA, cat# 12659) as a standard.

### 4.3. Immunofluorescence

To determine the oviductal localization of CHST10, the HNK-1 carbohydrate moiety (CHST10 product), ALDH9A1, ALDOA, and TUBB, we used unmated and mated female rats. For mating, we proceeded as described in Section 4.1.1; however, in this experiment, the organs were immediately processed as described previously [1]. The following antibodies were used for immunofluorescence detection: fluorescein isothiocyanate (FITC) mouse anti-HNK-1 (human CD57, BD Pharmingen, San Diego, CA, USA, cat# 555619) diluted 1:5; FITC mouse immunoglobulin M (IgM), k isotype control (BD Pharmingen, cat# 555583) diluted 1:5; rabbit anti-CHST10 polyclonal antibody (Sigma-Aldrich, cat# HPA012884) diluted 1:60; rabbit anti-ALDH9A1 polyclonal antibody (Invitrogen, Rockford, IL, USA, cat# PA5-52756) diluted 1:60; rabbit anti-ALDOA polyclonal antibody (Invitrogen, cat# PA5-77857) diluted 1:60; and mouse anti-TUBB monoclonal antibody (DSHB, Iowa City, IA, USA, cat# AB 2315513) diluted 1:100. To detect unconjugated primary antibodies, we used an anti-rabbit immunoglobulin G (IgG) Alexa Fluor^®^ 594 secondary antibody (Life Technologies, Eugene, OR, USA, cat# A21442) diluted 1:200 and an anti-mouse IgG IgM Alexa Fluor^®^ 488 secondary antibody (Life Technologies, cat# A10684) diluted 1:200. For the negative control group, the primary antibody incubation step was omitted. Slides were imaged using a Leica TCS SP8 confocal microscope (Leica Microsystems, Wetzlar, Germany).

### 4.4. Western Blotting

#### 4.4.1. Sample Preparation

The oviductal mucosa epithelial and stromal cell pellet was sonicated at power 20 for 10 s once in 200 μL of RIPA buffer (137 mM NaCl, 0.1% SDS, 0.5% sodium deoxycholate, 1% Triton X-100, 2 mM EDTA, 1× protease inhibitor cocktail and 20 mM Tris-HCl, pH 7.5), incubated at 4 °C for 30 min and sonicated again at power 20 for 10 s twice; subsequently, the sample was centrifuged at 8000× *g* for 10 min at 4 °C, and the supernatant was transferred to a new conical tube. This protein extract was considered the *cellular pool*. The total protein in the *cellular pool* was quantified using a DC protein assay kit II (Bio-Rad, cat# 500-0006) with BSA as a standard. The proteins were subsequently precipitated with cold acetone (5:1), resuspended in loading buffer to a concentration of 5 μg/μL, and stored at −20 °C until use.

The *secreted pool* of proteins from the oviductal mucosa collected in saline was concentrated from 400 to 25 µL for 1D electrophoresis using Microcon^®^-10 centrifugal filters (Merck Millipore Ltd., Carrigtwohill, IRL, cat# MRCPRT010). Afterward, the total protein content was quantified, and loading buffer was added to a final concentration of 5 μg/μL before storage at −20 °C. For 2D electrophoresis, the samples were concentrated from 400 to 10 μL because NaCl interferes with isoelectric focusing (IEF), mixed with 125 μL of C1 denaturing buffer (8 M urea, 1 M thiourea, 4% CHAPS, 66 mM DTT, 2 mM tributylphosphine and 0.8% ampholytes pH 3–10), and incubated for 2 h at room temperature with constant agitation. After centrifugation at 8000× *g* for 10 min, the supernatant was transferred to a new conical tube and immediately used for gel IEF.

#### 4.4.2. One-Dimensional Electrophoresis and Western Blotting

To detect changes in the levels of the CHST10, ALDH9A1, ALDOA, and HNK-1 glycoproteins in the oviductal mucosa induced by mating, we used unmated and mated female rats. The samples were resolved under reducing and denaturing conditions via sodium dodecyl sulfate–polyacrylamide gel electrophoresis (SDS-PAGE; 12%) and electroblotted onto nitrocellulose membranes (Bio-Rad, cat# 162-0115) overnight at 90 mA and 4 °C. After blocking for 1 h with 5% nonfat milk in PBSTr (137 mM NaCl, 2.7 mM KCl, 10.5 mM Na_2_HPO_4_, 1.8 mM KH_2_PO_4_, 0.2% Triton X-100), the membrane was incubated at 4 °C overnight with a rabbit anti-CHST10 polyclonal antibody diluted 1:1000, a mouse anti-HNK-1 monoclonal antibody (BD Pharmingen, cat# 559048) diluted 1:10,000, a rabbit anti-ALDH9A1 polyclonal antibody diluted 1:1000, or a rabbit anti-ALDOA polyclonal antibody diluted 1:3000 in PBSTr + 2% BSA. The membrane was subsequently washed and incubated with an anti-mouse IgG and IgM, horse radish peroxidase (HRP)-linked secondary antibody (Sigma-Aldrich, cat# AP130P) diluted 1:5000 or an anti-rabbit IgG, HRP-linked secondary antibody (Cell Signaling, Danvers, MA, USA, cat# 7074S) diluted 1:2000 for 1 h at room temperature. A rabbit anti-TUBB polyclonal antibody (Abcam, Cambridge, UK, cat# ab15568) diluted 1:5000 was used as a loading control for the *cellular pool* of proteins from the oviductal mucosa. Ponceau red or silver staining [55] of the Western blot membrane was used as a loading control for the *secreted pool* of proteins from the oviductal mucosa. The HRP conjugates were detected using Western Lightning Plus enhanced chemiluminescence substrate (PerkinElmer, Waltham, MA, USA, cat# NEL104001EA) and developed via Amersham Hyperfilm ECL (GE Healthcare, Little Chalfont, UK, cat# 28906836). The stained membranes and films were subsequently imaged using an OmniMedia Scanner (UMAX Data System, Hsinchu, Taiwan). Densitometry was performed using ImageJ version 1.54k software.

#### 4.4.3. Two-Dimensional Electrophoresis and Western Blotting

To identify the HNK-1 glycoproteins that are regulated by mating in the oviductal mucosa, we used unmated and mated females. A 7 cm immobilized pH gradient (IPG) strip (pH 3–10, Bio-Rad, cat# 163-2000) was rehydrated overnight under passive conditions with 125 μL of the *secreted pool* of proteins from the oviductal mucosa (350 μg in C1 denaturing buffer) for 12 h. Then, IEF was conducted in a linear range to a total of 3800 V-h at 20 °C with a current of 50 μA/IPG strip. Prior to SDS-PAGE, the gels were incubated with 10 mg/mL DTT in equilibration buffer (6 M urea, 30% glycerol, 2% SDS, 0.05 Tris-HCl, pH 8.8) for 15 min and then with 45 mg/mL iodoacetamide (IAA) in the same buffer for 15 min. SDS-PAGE was performed with 12% polyacrylamide gels (80 × 60 × 1.5 mm) and Tris–glycine–SDS buffer at 35 mA, followed by electroblotting onto a nitrocellulose membrane overnight at 90 mA and 4 °C. Western blotting was performed as previously described with the following modifications: the anti-HNK-1 mouse monoclonal antibody was diluted 1:2500, and the secondary antibody was diluted 1:30,000. After Western blotting, the membranes were stained with silver as described previously. Image analysis and spot matching were performed using Fiji/ImageJ version 2.9.0 software [56].

### 4.5. Protein Identification

#### 4.5.1. Destaining and Drying the Gel Pieces

The *secreted pool* of proteins from the oviductal mucosa was resolved via 2D electrophoresis and stained with Coomassie blue. The spot pattern was matched with the spot pattern obtained from the silver-stained membranes to identify the target proteins that were glycosylated with HNK-1 because of mating. The selected spots were excised with a scalpel, cut into small pieces, and destained with a destaining solution: 50 mM ammonium bicarbonate (AB) + 50% acetonitrile (ACN), overnight with shaking at room temperature (RT). The destained gel pieces were dried by washing twice in 100 mM AB for 10 min, twice with destaining solution for 10 min, and finally, with pure ACN for 10 min.

#### 4.5.2. Trypsin Digestion

The dried gel pieces were sent to the biOMICS MS facility (Sheffield University, UK). In the biOMICS laboratory, proteins in dry gel pieces were reduced on Cys residues by adding 50 μL of 50 mM Tris(2-carboxyethyl) phosphine and heating for 10 min at 70 °C, and the supernatant was removed and allowed to cool on the bench. The proteins were then alkylated on reduced Cys by adding 100 μL of 50 mM IAA and incubating for 30 min at RT in the dark. The following procedures were conducted at RT unless otherwise specified. The gel pieces were washed twice each step with shaking for 10 min with the following solutions: 200 μL of 100 mM AB; 100 μL of 50% ACN + 50 mM AB; and 100 μL of ACN. The supernatant was removed, and the samples were dried for 10 min. The proteins in the gel pieces were digested by adding 20 μL of 1 ng/μL trypsin (New England Biolabs, Ipswich, MA, USA, cat# P8101S) in 100 mM AB and incubating overnight at 37 °C. The digestion was stopped by adding 10 μL of ACN to the gel pieces and incubating them for 15 min at 37 °C. The resulting supernatant, containing the digested peptides, was transferred into a new conical tube. The digested peptides were extracted twice from gel pieces with 25 μL of 0.5% formic acid (FA) and incubated for 15 min with shaking, followed by the addition of 50 μL of ACN and incubation for 15 min with shaking. The final extraction was conducted by adding 50 μL of ACN to the gel pieces and incubating them for 15 min with shaking. The supernatants were transferred into the same conical tube, and the total volume of extracted peptides (230 μL) was dried in a speed-vac for 3 h. Then, the dry pellet of each sample was resuspended by sonication in a water bath for 2 min at a power of 9 in 40 μL of 0.5% FA, after which 20 μL was transferred into an MS vial for further analysis.

#### 4.5.3. Liquid Chromatography and Mass Spectrometry Analysis

The trypsin-digested peptides in the MS vials were separated and analyzed using an HPLC UltiMate 3000 coupled with an Orbitrap Elite™ Hybrid Ion Trap-Orbitrap mass spectrometer (Thermo Fisher Scientific, Waltham, MA, USA). For high-performance liquid chromatography, the sample injection volume was 18 μL, the flow rate was 0.250 μL/min, solvent A was 0.1% FA, and solvent B was 0.1% FA + 80% ACN. The gradient was as follows: 3% solvent B for 10 min, 40% solvent B for 40 min, 90% solvent B for 5 min, and 90% solvent B for 5 min. Solvent A was used to complete 100% of the total solvent in each step. For MS analysis in the Orbitrap, the operation mode was CID with minimal signal: 5000; normalized collision energy: 35; default charge state: 2; charge state: ≥ 2+; ion injection time: 30 milliseconds; scan range: 375–1600 *m*/*z*; and top 20 ions.

#### 4.5.4. Criteria for Protein Identification

Protein identification was performed using MaxQuant (v.1.5.5.1) for each sample individually using default parameters with the *Rattus norvegicus* database (Uniprot_Proteome_RattusNorvegicus_CanoIso_2017-07-24.fasta) and the reverted database to determine the false discovery rate (FDR). Trypsin/P was specified as the cleavage enzyme, allowing up to 2 missing cleavages. The mass tolerance for precursor ions was set as 20 ppm. Carbamidomethyl on cysteine was specified as a fixed modification, whereas protein N-terminal acetylation and methionine oxidation were set as variable modifications. The FDR was adjusted to a 1% decoy reverted database.

### 4.6. Relative Gene Expression Levels

RT-qPCR was used to compare the expression levels of *Chst10* in oviductal mucosa samples from the VCS and sham control groups and among the saline, sperm, and SVF groups. *Actb* and *Gapdh* were used as reference genes to calculate the relative gene expression levels. Information regarding the total RNA isolation procedure, primer sequences, RT-qPCR conditions, and data analyses was reported previously [1].

### 4.7. Statistical Analysis

The Mann–Whitney test was used to determine the significance of the differences in the various measured factors between unmated and mated rats. The Kruskal-Wallis test followed by the uncorrected Dunn test was used to determine the significance of the differences in the RT-qPCR results among the saline, sperm, and SVF groups (Figure 10). The lines in the plots represent the median and interquartile range (IQR) of the corresponding dataset. * *p* < 0.05, ** *p* < 0.01, and *** *p* < 0.001 were considered to indicate significant differences. All the statistical analyses were conducted using GraphPad Prism 6 version 6.04 software (GraphPad, Boston, MA, USA).

## 5. Conclusions

Mating increases the activity of CHST10 in the oviductal mucosa, which in turn induces the synthesis of acidic variants of ALDH9A1 and FHL1 via HNK-1 glycosylation. ALDH9A1, HNK-1-ALDH9A1, and/or other HNK-1 glycoproteins could participate in the negative selection of sperm in the UTJ, since we detected acrosome-reacted sperm apparently interacting with regions where these proteins are located. Finally, the sensorial component of mating could regulate early events (e.g., sperm transport and selection) occurring in the oviductal mucosa after mating.

## Figures and Tables

**Table 1:** Proteins identified by mass spectrometry (MS).

Spot Number	Protein	Gene	Sequence Coverage (%)	Molecular Mass (kDa)
2	Aldehyde dehydrogenase 9 family, member A1	*Aldh9a1*	37.2	54.1
3	Aldehyde dehydrogenase 9 family, member A1	*Aldh9a1*	45.3	54.1
4	Fructose bisphosphate aldolase A	*Aldoa*	42.0	39.4
5	Four and a half LIM domains protein 1	*Fhl1*	59.3	31.9
6	Four and a half LIM domains protein 1	*Fhl1*	68.2	31.9

biOMICS MS facility (Sheffield University, UK).

## Data Availability

The original contributions presented in this study are included in the article/Supplementary Material. Further inquiries can be directed to the corresponding author.

## Supplementary Materials

The following supporting information can be downloaded https://www.mdpi.com/article/10.3390/ijms26073309/s1.
