# Supplementary material for: Mating Increases CHST10 Activity in Rat Oviductal Mucosa to Induce the Synthesis of HNK-1 Glycoproteins: Possible Role in Sperm–Oviduct Interactions
Source: Int J Mol Sci. 2025 Apr 2;26(7):3309. doi: 10.3390/ijms26073309 (PMC11989750; doi:10.3390/ijms26073309)
Supplement: Supplementary file 1 [file ijms-26-03309-s001.zip › ijms-3461854-supplementary.pdf]

Figure S1:

Expression of the HNK-1 carbohydrate moiety and ALDH9A1 in sperm located in the utero-tubal junction (UTJ). The HNK-1 moiety and ALDH9A1 were detected via immunofluorescence. In all panels, blue fluorescence: Hoechst nuclear staining; red fluorescence: ALDH9A1; and green fluorescence: HNK-1 moiety. Inset rectangles in the upper panel were zoomed and displayed in the bottom panels A and B. (A) Acrosome-reacted sperm apparently interacted with regions in which ALDH9A1 and HNK-1 signals overlap. Three sperm tails and one sperm head can be observed. One tail seems to be connected to the unique sperm head (white arrowhead). (B) Acrosome-reacted sperm apparently crossing the oviductal mucosa. Several entangled sperm tails are located in the trenches of the continuous longitudinal folds. One tail seems to be connected to the unique sperm head (white arrowhead). In both, A and B, HNK-1 is located on the middle piece of the sperm tail (green arrows), and ALDH9A1 is located into the middle piece of the sperm tail (red arrows). Original magnification 630× (scale bar, 25 μm).

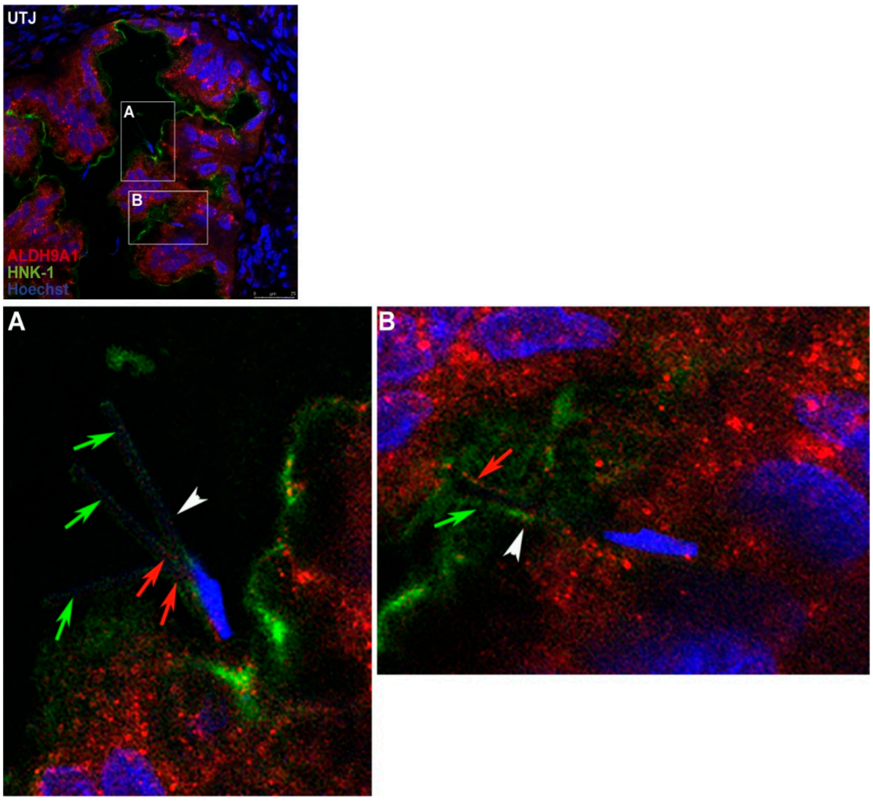

Figure S2: Expression patterns of the Tubulin-β in the rat oviduct. TUBB were detected via immunofluorescence. In all panels, blue fluorescence: Hoechst nuclear staining; and green fluorescence: TUBB. The square in the middle panel is the location of the magnified image displayed in the right panel. Green arrows: ciliated cell clusters in the trenches of the mucosal folds. Original magnification 200× (scale bar, 25 μm). The image in the right panel is shown at a magnification of 630× (scale bar, 10 μm).

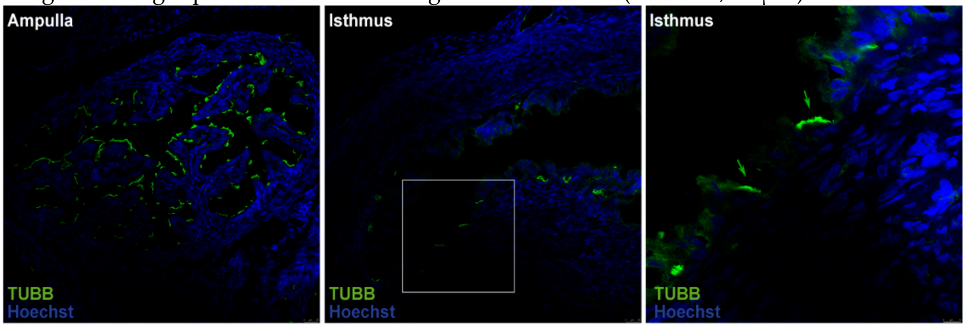

Figure S3:
Sequence of the human antigen used to raise the antibody anti-CHST10 (Sigma–Aldrich, cat# HPA012884, Prestige Antibodies).

Recombinant Protein Epitope Signature Tag (PrEST) antigen sequence:

PEVRKLPEEKHIPEELKPTGKELPDSQLVQPLVYMERLELIRNVCRDDAL

KNLSHTPVSKFVLDRIFVCDKHKILFCQTPKVGNTQWKKVLIVLNGAFSS

IEEIPENVVHDHEKNGLPRLSSFSDAEIQKRL

Figure S4A:
Sequence identity between antigen sequence for human CHST10 with rat CHST10 isoform X1.

Carbohydrate sulfotransferase 10 isoform X1 [Rattus norvegicus]

NCBI Reference Sequence: XP_006244792.1

Sequence ID: Query_465195Length: 374Number of Matches: 1

Range 1: 66 to 197Graphics

Alignment statistics for match #1

Score	Expect	Method	Identities	Positives	Gaps
216 bits(549)	3e-75	Compositional matrix adjust.	107/132(81%)	114/132(86%)	0/132(0%)

```
Query  1    PEVRKLPEEKHIPEELKPTGKELPDSQLVQPLVYMERLELIRNVCRDDALKNLSHTPVSK  60
            PE  KL  EKH  E +KPTGK L +S   QP VY+ERLELIRN C+++AL+NLSHT VSK
Sbjct  66   PEAEKLRGEKHFSEVMKPTGKMLSESHPDQPPVYLERLELIRNACKEEALRNLSHTEVSK  125

Query  61   FVLDRIFVCDKHKILFCQTPKVGNTQWKKVLIVLNGAFSSIEEIPENVVHDHEKNGLPRL  120
            FVLDRIFVCDKHKILFCQTPKVGNTQWKKVLIVLNGAFSSIEEIPENVVHDHEKNGLPRL
Sbjct  126  FVLDRIFVCDKHKILFCQTPKVGNTQWKKVLIVLNGAFSSIEEIPENVVHDHEKNGLPRL  185

Query  121  SSFSDAEIQKRL  132
            SSFS   IQKRL
Sbjct  186  SSFSKIGIQKRL  197
```

Figure S4B:
Sequence identity between antigen sequence for human CHST10 with rat CHST10 isoform X2.

Carbohydrate sulfotransferase 10 isoform X2 [Rattus norvegicus]

NCBI Reference Sequence: XP_017451740.1

Sequence ID: Query_7009531Length: 356Number of Matches: 1

Range 1: 48 to 179Graphics

Alignment statistics for match #1

Score	Expect	Method	Identities	Positives	Gaps
216 bits(549)	2e-75	Compositional matrix adjust.	107/132(81%)	114/132(86%)	0/132(0%)

```
Query  1    PEVRKLPEEKHIPEELKPTGKELPDSQLVQPLVYMERLELIRNVCRDDALKNLSHTPVSK  60
            PE  KL  EKH  E +KPTGK L +S   QP VY+ERLELIRN C+++AL+NLSHT VSK
Sbjct  48   PEAEKLRGEKHFSEVMKPTGKMLSESHPDQPPVYLERLELIRNACKEEALRNLSHTEVSK  107

Query  61   FVLDRIFVCDKHKILFCQTPKVGNTQWKKVLIVLNGAFSSIEEIPENVVHDHEKNGLPRL  120
            FVLDRIFVCDKHKILFCQTPKVGNTQWKKVLIVLNGAFSSIEEIPENVVHDHEKNGLPRL
Sbjct  108  FVLDRIFVCDKHKILFCQTPKVGNTQWKKVLIVLNGAFSSIEEIPENVVHDHEKNGLPRL  167

Query  121  SSFSDAEIQKRL  132
            SSFS   IQKRL
Sbjct  168  SSFSKIGIQKRL  179
```

Figure S4C:
Sequence identity between antigen sequence for human CHST10 with rat CHST10 isoform X3.

Carbohydrate sulfotransferase 10 isoform X3 [Rattus norvegicus]

NCBI Reference Sequence: XP_038938897.1

Sequence ID: Query_91391Length: 288Number of Matches: 1

Range 1: 2 to 111Graphics

Alignment statistics for match #1

Score	Expect	Method	Identities	Positives	Gaps
191 bits(486)	7e-67	Compositional matrix adjust.	94/110(85%)	100/110(90%)	0/110(0%)

```
Query  23   LPDSQLVQPLVYMERLELIRNVCRDDALKNLSHTPVSKFVLDRIFVCDKHKILFCQTPKV  82
            L +S   QP VY+ERLELIRN C+++AL+NLSHT VSKFVLDRIFVCDKHKILFCQTPKV
Sbjct  2    LSESHPDQPPVYLERLELIRNACKEEALRNLSHTEVSKFVLDRIFVCDKHKILFCQTPKV  61

Query  83   GNTQWKKVLIVLNGAFSSIEEIPENVVHDHEKNGLPRLSSFSDAEIQKRL  132
            GNTQWKKVLIVLNGAFSSIEEIPENVVHDHEKNGLPRLSSFS   IQKRL
Sbjct  62   GNTQWKKVLIVLNGAFSSIEEIPENVVHDHEKNGLPRLSSFSKIGIQKRL  111
```
